# P127: Hand hygiene survey: a prospective, interventional study of current practices in a multicentre regional hospital

**DOI:** 10.1186/2047-2994-2-S1-P127

**Published:** 2013-06-20

**Authors:** O Clerc, P Deriaz, B Duvillard, P Vanderavero, P Erard

**Affiliations:** 1 Hôpital Neuchâtelois, Site de Pourtalès, 2000 Neuchâtel, Switzerland

## Introduction

Hand hygiene (HH) compliance is of paramount importance to prevent infections in hospital settings.

## Objectives

We started an 18 months project of monitoring and improvement of HH in September 2012. We aimed to modify behaviours in order to obtain a minimal compliance of 80% by the end of the project.

## Methods

All professionals in direct contact with patients are planned to be evaluated during 8 rounds of observations. Compliance with the five indications of HH as defined by the World Health Organisation is rated by direct observation by trained personnel. Excessive indications are also quoted. An immediate feed-back is transmitted to the observed professionals. Results are provided to each unit by professional category, and units are secondarily ranked. Restitution of results occurs using a dedicated journal that is sent to all hospital collaborators. Other communication and training tools are sequentially developed. Infection control unit is available on request for training interventions in units, with priority for the ones showing the lowest compliance.

## Results

Each audit evaluated around 3000 indications for HH in 51 units. The first round of observations showed a general compliance with HH indications of 61%, ranging globally from 48% to 69% depending on heath-care workers categories. Extremes measured for individuals varied between 6 and 100%. The intervention was well-accepted in the hospital, and unsatisfying results resulted in spontaneous requests for training in 12 units (24% of all units). Second round of observations showed a global improvement of compliance with HH to 73%.

## Conclusion

Short term evaluation of an ongoing prospective, interventional study of hand hygiene evaluation using restitution of results as primary mean of intervention showed a significant increase in the compliance with HH in a multicentre, regional hospital. Persistence of this improvement and active participation of caregivers as a marker of greater awareness to HH will be prospectively monitored.

## Disclosure of interest

None declared

